# Effect of exposure of teeth to Ph change of chlorinated water on shear bond strength of metal orthodontic brackets (an in vitro study)

**DOI:** 10.1186/s12903-025-06100-4

**Published:** 2025-05-15

**Authors:** Eman A. Mustafa, Seham A. Hanafy, Tarek N. Yousry, Hanan A. Ismail

**Affiliations:** 1 Orthodontic Department, Faculty of Dentistry, Alexandria University, Alexandria, Egypt; 2 Faculty of Dentistry, Alexandria University, Alexandria, Egypt; 3 Faculty of Dentistry, Alexandria University, Pharos University, Alexandria, Egypt

**Keywords:** Chlorinated water, Enamel erosion, Orthodontic bracket bonding, Shear bond strength

## Abstract

**Background:**

Swimming is widely recognized as one of the healthiest forms of exercise, but chlorinated water in swimming pools can adversely affect orthodontic bracket bonding. The objective of this study was to determine the effects of chlorinated water with varying pH levels on the shear bond strength (SBS) of metal brackets and to assess the adhesive remnant index (ARI) following bracket debonding.

**Materials and methods:**

A total of 126 sound premolars (71 maxillary and 55 mandibular) were randomly divided into three experimental groups: two test groups and one control group. In the test groups (Groups 1 and 2), the teeth were soaked in chlorinated water at two different pH values (pH 7.4 and pH 3), whereas in the control group (Group 3), the teeth were soaked in artificial saliva (pH 7). The soaking period lasted for 12 days to simulate one year of swimming training. A consistent bonding protocol was applied for all the samples. Each group was further randomly divided into three subgroups of 14 premolars to compare the SBS and ARI values immediately after bonding (Subgroup 1), after 6 days of bonding (mimicking 6 months of training) (Subgroup 2), and after 12 days of bonding (mimicking one year of training) (Subgroup 3).

**Results:**

In all the subgroups, the control group consistently had a higher SBS than did the test groups. Compared with Group 1, Group 2 had a significantly lower SBS. Specifically, the means and standard deviations in Group 1 were 7.34 ± 0.99 MPa for Subgroup 1, 6.89 ± 0.95 MPa for Subgroup 2, and 5.59 ± 1.09 MPa for Subgroup 3. In Group 2, the values were 6.12 ± 0.72 MPa for Subgroup 1, 5.82 ± 0.70 MPa for Subgroup 2, and 4.52 ± 0.86 MPa for Subgroup 3. Conversely, Group 3 presented means and standard deviations of 9.01 ± 0.99 MPa for Subgroup 1, 9.06 ± 0.91 MPa for Subgroup 2, and 9.10 ± 0.92 MPa for Subgroup 3. The ARI values were not significantly different between the groups.

**Conclusion:**

The pH of chlorinated swimming pool water affects the bond strength of orthodontic brackets, with a more acidic pH resulting in diminished bond strength. Accordingly, continuous monitoring of the pH of swimming pool water is essential.

## Background

Swimming is recognized as one of the most popular activities that offers a comprehensive workup for the entire body. However, professional swimmers may experience some side effects [1], particularly concerning their dental health. These side effects include dental erosion, calculus formation, and tooth staining [2].

Chlorine is commonly used in swimming pools to disinfect water and eliminate contaminants and harmful microorganisms. According to the Centers for Disease Control and Prevention (CDC), the recommended chlorine concentration for pool disinfection is between 1 and 3 parts per million (ppm), while the pH of the water should be maintained between 7.2 and 7.8. Continuous monitoring of these parameters is essential to mitigate the adverse effects of chlorinated water on swimmers [3]. When chlorine interacts with water, it forms hypochlorous acid and hypochlorite ions. These compounds lead to a decrease in pH, rendering the pool water more acidic. To rectify this, buffers such as soda ash are utilized to restore the pH to the appropriate range [4].

Research by Abdelrahman et al. [2] has demonstrated a significant prevalence of dental erosion among competitive and recreational swimmers who have been trained for approximately six hours per week. These findings indicate that chlorinated water, owing to its acidic nature and insufficient monitoring practices, contributes to the erosion of dental structures. Furthermore, Buczkowska et al. noted that if adequate amounts of soda ash are not used to buffer pool water, the pH can decrease to 3, a condition that poses a risk for tooth erosion [4].

Dental erosion is defined as the loss of tooth structure resulting from acid dissolution, independent of bacterial involvement [5]. Its prevalence tends to increase with age [6] and is influenced by various biological and behavioral factors. These factors enable acids to interact with the enamel, leading to gradual dissolution until the dentin is exposed [3]. Poorly monitored swimming pools are defined as acidic environments that contribute to this phenomenon [2].

The bonding of orthodontic brackets is a critical phase in orthodontic treatment, with a minimal bond strength requirement of 6_8 MPa for most clinical applications [7]. Several factors influence bond strength, including enamel properties, the adhesive layer, the type of bracket used, the characteristics of the oral cavity, and masticatory forces [8]. Healthy enamel is mandatory for achieving optimal bond strength with orthodontic brackets; therefore, enamel erosion adversely affects this bond. The literature on this topic shows conflicting results. Some studies [9–11] reported no significant difference in SBS between healthy and eroded enamel, whereas another study [12] reported an increase in bond strength with eroded enamel. Additionally, two studies [13, 14] reported a decrease in the bond strength under similar conditions.

In orthodontics, the adhesive remnant index (ARI) is a widely used system for assessing the amount of adhesive remaining on the tooth following bracket debonding [15], thus facilitating an accurate determination of the level of bond failure [16]. The majority of existing studies [9–14] have focused on dental erosion caused by exposure to acidic beverages. To our knowledge, no research has previously investigated the bond strength of orthodontic brackets to enamel that has been eroded by chlorinated water.

Consequently, our objective in this study was to evaluate the impact of chlorinated water with different pH values on the SBS of metal orthodontic brackets. The null hypothesis suggests that there is no significant difference in SBS between teeth soaked in chlorinated water at pH 7.4 and pH 3 (test groups) and teeth soaked in artificial saliva with a pH of 7 (control group).

## Materials and methods

A randomized controlled in vitro study was conducted to evaluate the shear bond strength (SBS) and adhesive remnant index (ARI) of metal orthodontic brackets bonded to teeth soaked in three different solutions: artificial saliva (control), chlorinated water with a pH of 7.4, and chlorinated water with a pH of 3.

The research protocol received approval from the institutional review board of the Faculty of Dentistry, Alexandria University (IRB: 00010556–IORG: 0008839). All the methods followed CRIS guidelines and regulations [17]. The study setting was implemented in the Orthodontic Department and the Dental Biomaterials Department at Alexandria University, Egypt.

### Sample grouping and preparation

The required sample size was determined on the basis of an alpha level of 0.05 and a power of 0.8. Previous studies reported a mean ± SD shear bond strength of 10.70 ± 2.24 MPa for neutral saliva [18], whereas the mean bond strength was 13.09 ± 1.90 MPa for distilled water and 6.04 ± 1.11 MPa for the Coca-Cola beverage [19]. It was hypothesized that distilled water would exhibit effects similar to those of neutral swimming pool water, whereas Coca-Cola, with a pH of 2.5, would reflect the detrimental effect of a highly acidic environment on tooth enamel. To ensure adequate study power, the sample density was calculated on the basis of differences between neutral saliva and distilled water, resulting in a requirement of 13 samples per group. This was adjusted to 14 samples per group to account for potential laboratory processing errors, leading to a total sample size of 126 samples. The calculation was conducted via G* Power software (V. 3.1.9.7) [20].

A total of 126 sound human premolar teeth (71 maxillary and 55 mandibular) were collected, all of which were freshly extracted for orthodontic reasons. Visual examination confirmed the absence of obvious cracks or decalcification in the teeth. The teeth were cleaned under tap water, pumiced, and subsequently stored in saline (0.9% NaCl) solution, with weekly changes until the commencement of the study [21].

### Sample allocation

Upon initiation of the experiment, each tooth was assigned a unique identification number from 1 to 126. The teeth were then randomly allocated into three experimental groups via a random number generator.

### Experimental methods (Fig. 1)

**Fig. 1:**
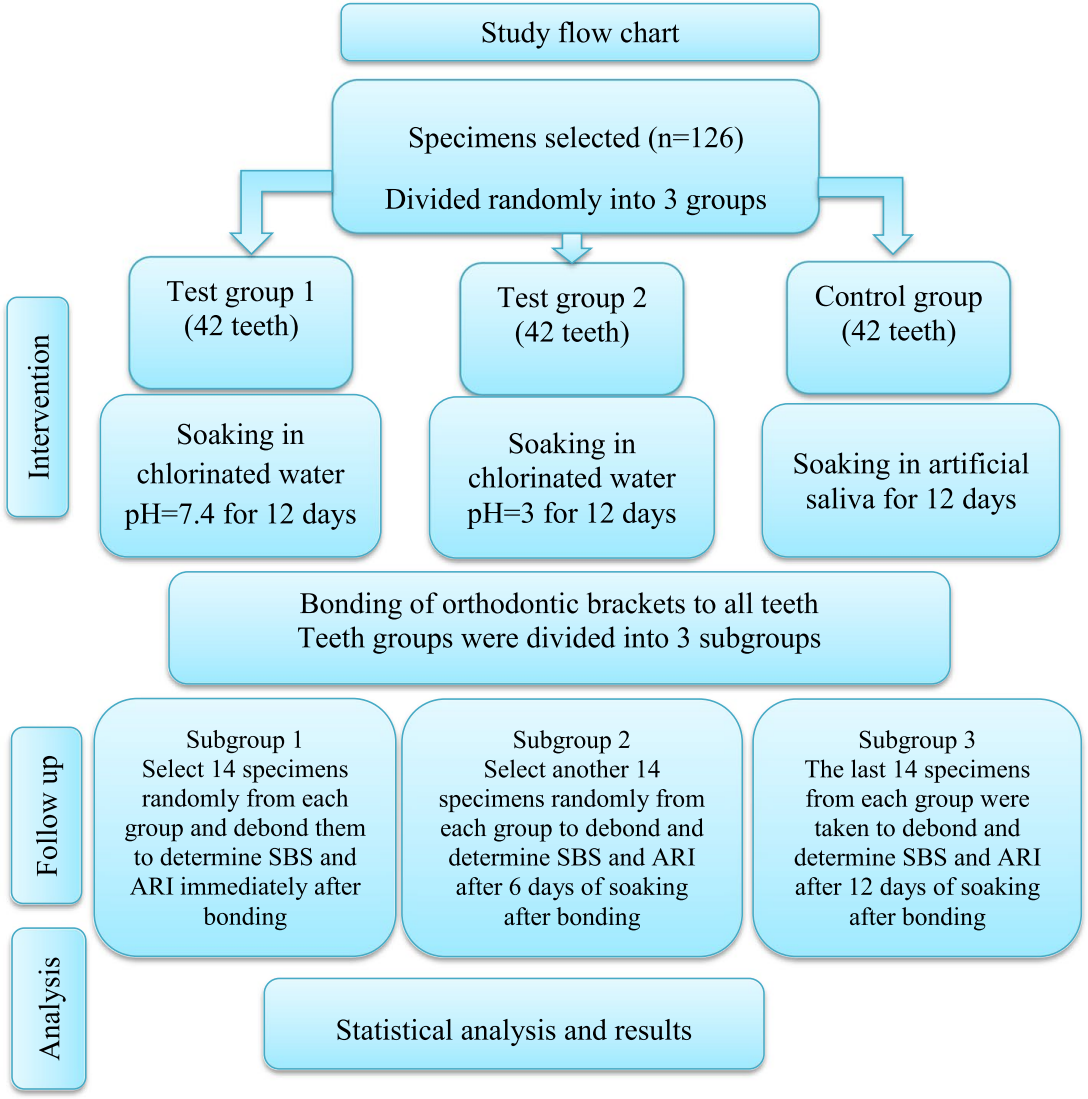
Flow chart of the study

Group 1 (test): forty-two teeth were soaked in chlorinated water with a pH of 7.4 [3].

For Group 2 (test), 42 teeth were soaked in chlorinated water with a pH of 3 [3].

Group 3 (control): forty-two teeth were soaked in artificial saliva (20 mmol/L CaCl_2_, at neutral pH 7).

The composition of the saliva was designed to simulate the clinical conditions and is detailed in Table 1. The artificial saliva was maintained at room temperature (37 °C) and neutral pH to accurately replicate the oral environment. The inclusion of calcium in artificial saliva renders it more representative of physiological conditions than formulations devoid of calcium [22].

**Table 1:** Composition of artificial saliva and quantities

Sorbitol	**30.0 g**
Potassium chloride	**625 mg**
Calcium chloride	**166 mg**
Magnesium chloride	**59 mg**
Dipotassiummonohydrogen phosphate	**804 mg**
Potassium dihydrogen phosphate	**366 mg**
Carboxymethylcellulose sodium	**11.7 g**
Purified water	**Add 1000 ml**

The pH of the solution was monitored daily via a Lovibond water testing device (MD200 photometer 5 in 1, Tintometer Group, Germany), which detects chlorine levels and the pH of the water.

### Preparation of chlorinated water

Three labeled containers were used for soaking the teeth. The first container contained chlorinated water with a pH of 7.4, which was prepared by adding 90% trichloroisocyanuric acid (TCCA 90% powder, swimming pool chlorine, China) to tap water. The water was tested via a Lovibond water testing device, and the chlorine concentrations were adjusted to between 1 and 3 ppm. The pH was set to 7.4 using hydrochloric acid (HCl 33%, Diamond, China) [3]. The second container was prepared similarly, with the pH adjusted to 3 [4]. The third container contained artificial saliva, reflecting a neutral pH of 7 and comprising 20 mmol/L CaCl_2_. The chlorinated water was prepared daily to ensure consistent conditions.

Assuming that, on average, a swimming trainee engages in approximately 6 h of training per week [2], this equates to 24 h (one day) of training in one month. Consequently, the soaking times for the teeth were adjusted to reflect this schedule.

Following 12 days of soaking _simulating one year of swimming training_ brackets were bonded to all the teeth.

### Bonding protocol [21]

Thirty-seven percent phosphoric acid (Meta Etchant, Meta Biomed, Korea) was applied to the teeth for 30 s, followed by rinsing for a minimum of 5 s. The teeth were then dried using oil-free air until a chalky white appearance was achieved. The light-cured bond(Ortho Solo Universal Sealant and Bond Enhancer, Ormco Corp, Glendora, California, USA) was meticulously applied with a microbrush and subsequently dried with oil-free air. Grengloo adhesive (Ormco, Glendora, California, USA) was utilized for bracket application(Ormco, Mini 2000, Ormco Corp, Glendora, California, USA). The brackets were carefully positioned at the center of the buccal surface and pressed firmly to allow for the removal of any excess adhesive. Curing was performed on both the mesial and distal surfaces for 20 s with a light-curing device (Woodpecker i-led, 2300 mW/cm^2^; Woodpecker, China). All bonding procedures adhered strictly to the manufacturer's guidelines.

For each tooth, a chemically cured acrylic resin cylindrical mold was created, ensuring that the buccal surface of each tooth remained perpendicular to the mold's base, which was verified via a surveyor. The molds were subsequently placed in a common container.

During the 1-year simulations to replicate the oral environment, all samples were subjected to thermocycling (SD Mechatronik, Feldkirchen-Westerham, Germany). A total of 10,000 thermocycles were completed in water, fluctuating between 5 °C and 55 °C, with a dwell time of 30 s and a transfer time of 5 s [23].

The samples were categorized into three subgroups to facilitate a comparison of SBSs and ARIs postdebonding. Each group consisted of fourteen specimens tested immediately after bonding (Subgroup 1), after 6 days of bonding (which simulates 6 months of swimming training) (Subgroup 2), and after 12 days of bonding (to mimic one year of swimming training) (Subgroup 3).

### Shear Bond Strength (SBS)

**Fig. 2:**
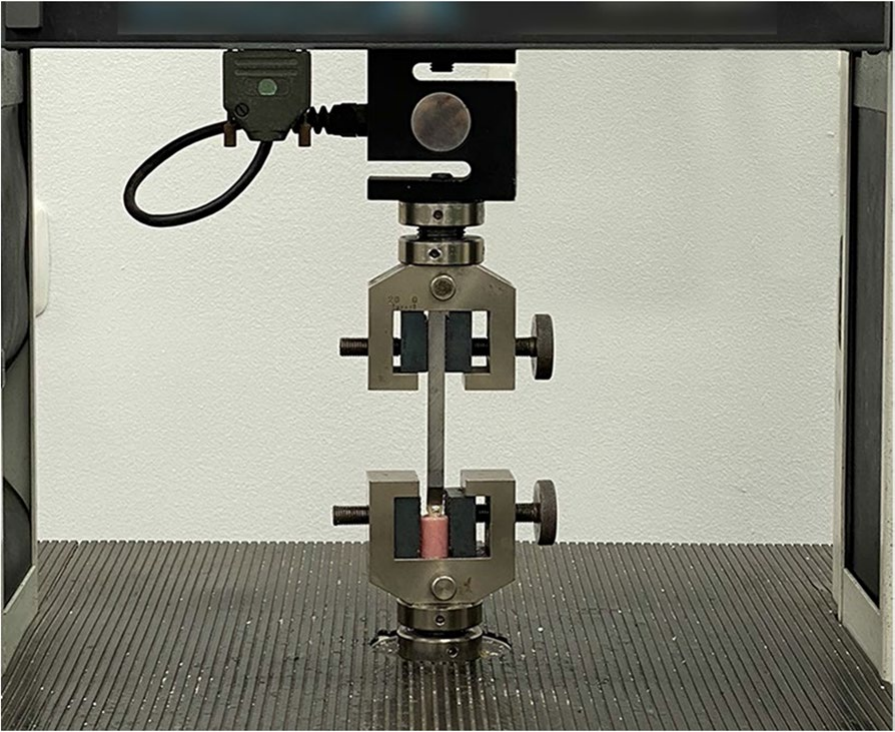
Universal testing machine

SBS tests were executed via a universal testing machine (LR 5 K Lloyd, UK) (Fig. 2). The cross-head speed was meticulously adjusted to 0.5 mm/min, with the tooth securely fixed in a holding ring on the machine’s lower table. The tapered blade of the machine applied force between the bracket base and the tooth, recording the debonding force in Newtons on a display monitor. The registered measurements were subsequently converted to megapascals (MPa) by dividing the force by the bracket base area to determine the bond strength.

### Adhesive Remnant Index (ARI)(Fig. 3)

**Fig. 3:**
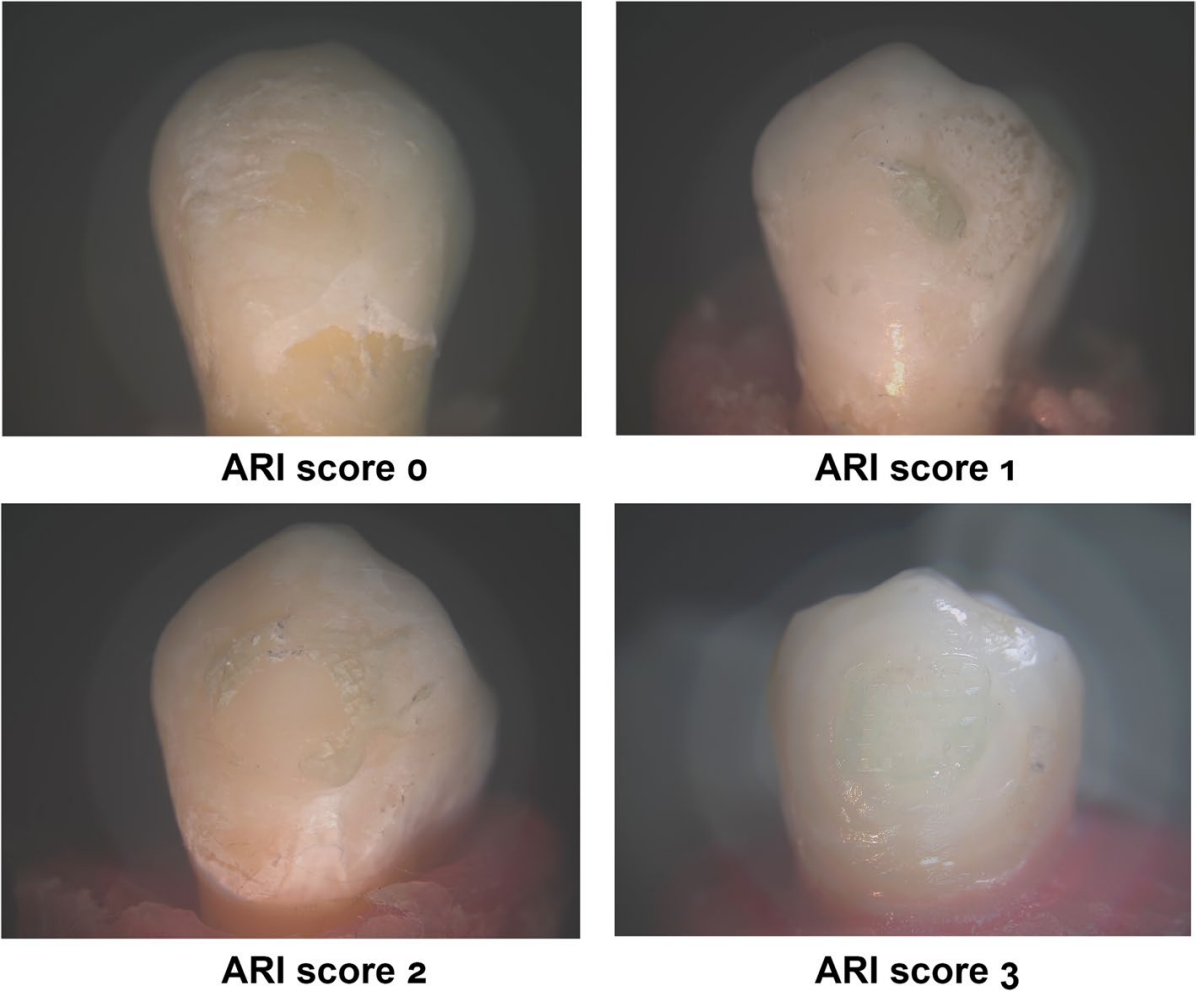
20 × magnification of the stereomicroscope used to determine the ARI

An optical stereomicroscope (Olympus SZ-CTV, Japan) was employed to evaluate the quantity of adhesive remaining on the tooth surface following debonding. This assessment involved determining the amount of adhesive remaining on the enamel surface at 20X magnification, accompanied by digital photographs of each tooth. Each tooth received a score from 0 to 3, as outlined by Artun and Bergland [24].

- Score 0: No adhesive remains on the tooth surface; bond failure occurred entirely at the resin_enamel interface.
- Score 1: Less than half of the adhesive remains on the tooth surface; bond failure occurs more frequently at the resin_enamel interface.
- Score 2: More than half of the adhesive remains on the tooth surface; bond failure occurs predominantly at the resin_enamel interface.
- Score 3: the entire adhesive remains on the tooth surface; bond failure is entirely at the interface between the bracket and the resin.

### Statistical analysis

Data collection and analysis were conducted via the Statistical Package for Social Science (IBM SPSS, version 23; Armonk, NY, USA) [25]. The normality of the SBS and the ARI was assessed via the Shapiro_Wilk test and Q_Q plots. A homogeneous distribution was identified for the SBS, whereas the ARI exhibited a nonhomogeneous distribution. Data were summarized using the mean and standard deviation (SD), in addition to the median, minimum, and maximum values. Furthermore, the distributions of the ARI scores are presented as frequencies and percentages. The Kruskal_Wallis test, accompanied by Dunn’s post hoc test, was employed to evaluate the ARIs among different groups and time points. All the statistical tests were two-tailed, and the significance level was established at a P- value < 0.05.

## Results

**Fig. 4:**
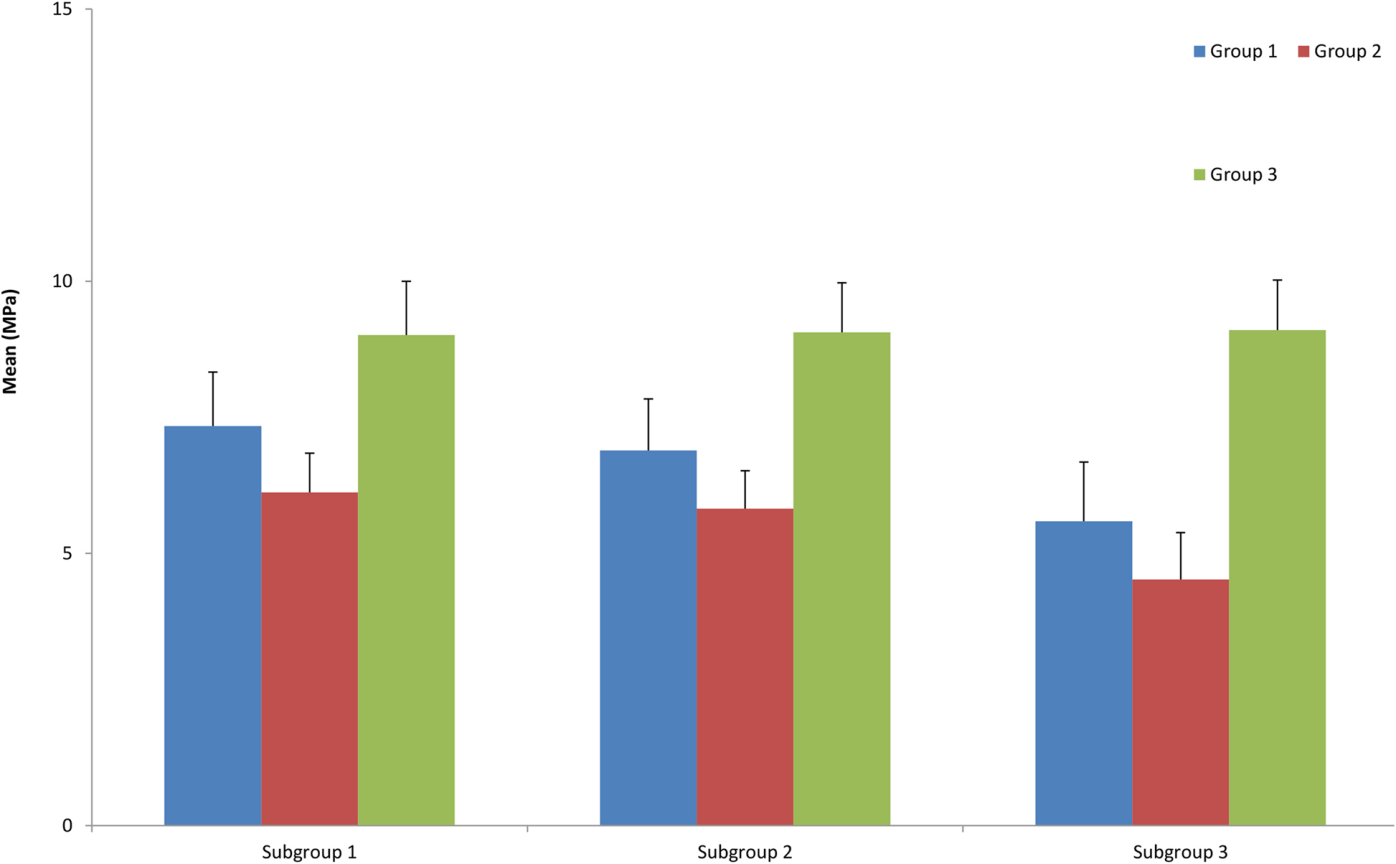
Comparison of the means and standard deviations of the SBSs between the test and control groups

Table 2: Across all time points, Group 3 displayed the highest mean, median, and minimum–maximum values of SBS when compared to Groups 1 and 2, with Group 2 exhibiting the lowest values. The results consistently indicate that more acidic conditions are correlated with significantly lower SBSs across all the evaluated time points. (Fig. 4).

**Table 2:** Comparison of SBS, in MPa, among the study groups. Group 1: test (chlorinated water at pH 7.4); Group 2: test (chlorinated water at pH 3); Group 3: control (artificial saliva at pH 7). Each subgroup comprises 14 teeth from each group tested at different time points. Subgroup 1: (immediately after bonding), Subgroup 2: (after 6 days of bonding), Subgroup 3: (after 12 days of bonding)

**Time Points**		**Group 1** **(***n*** = 42)**	**Group 2** **(***n*** = 42)**	**Group 3** **(***n*** = 42)**	***P-*value**
Subgroup 1	Mean ± SD	7.34 ± 0.99	6.12 ± 0.72	9.01 ± 0.99	< 0.0001*
	Median	7.35	6.33	8.83	
	Min – Max	5.78–9.35	5.00–7.00	7.55–10.44	
Subgroup 2	Mean ± SD	6.89 ± 0.95	5.82 ± 0.70	9.06 ± 0.91	< 0.0001*
	Median	6.95	6.09	9.04	
	Min – Max	5.53–8.04	4.51–6.58	7.72–10.60	
Subgroup 3	Mean ± SD	5.59 ± 1.09	4.52 ± 0.86	9.10 ± 0.92	< 0.0001*
	Median	5.89	4.74	9.20	
	Min – Max	4.00–7.16	3.42–5.91	7.47–10.25	
***P* value**		< 0.001*	< 0.001*		0.972

^*^Statistics are significant at a *p*- value < 0.05

Table 3: Presenting the mean, median, and (minimum_maximum) values of the ARIs for the study groups across all time points. The P- value did not significantly differ between the study groups at any time point.

**Table 3:** Comparison of the ARIs between the study groups. Group 1: test (chlorinated water at pH 7.4); Group 2: test (chlorinated water at pH 3); Group 3: control (artificial saliva at pH 7). Each subgroup comprises 14 teeth from each group tested at different time points. Subgroup 1: (immediately after bonding), Subgroup 2: (after 6 days of bonding), Subgroup 3: (after 12 days of bonding)

Time Points		**Group 1 (***n*** = 42)**	**Group 2 (***n*** = 42)**	**Group 3 (***n*** = 42)**	***P-*value**^**1**^
Subgroup 1	Mean ± SD	1.29 ± 0.83	1.50 ± 0.76	1.50 ± 0.76	0.681
	Median	1.00	1.50	1.50	
	Min – Max	0.00–3.00	0.00–3.00	0.00–3.00	
Subgroup 2	Mean ± SD	1.36 ± 0.63	1.57 ± 0.85	1.36 ± 0.63	0.908
	Median	1.00	1.50	1.00	
	Min – Max	0.00–2.00	0.00–3.00	0.00–2.00	
Subgroup 3	Mean ± SD	1.57 ± 0.76	1.93 ± 0.92	1.43 ± 0.85	0.301
	Median	1.00	2.00	1.50	
	Min – Max	1.00–3.00	0.00–3.00	0.00–3.00	
**P value**^**1**^		0.301	0.906		0.909

^*^Statistics are significant at a P- value < 0.05, P value^1^: Kruskal_Wallis test

Table 4: In Subgroup 1, the most common ARI scores were 1 and 2 for Groups 3 and 2, respectively, whereas, in Group 1 the most common score was 1. In Subgroup 2, a score of 1 was the most frequent across all groups. In Subgroup 3, the most common ARI score was 2 for Groups 3 and 2, whereas Group 1 mostly had a score of 1.

**Table 4:** Distribution of ARIs among the study groups. Group 1: test (chlorinated water at pH 7.4); Group 2: test (chlorinated water at pH 3); Group 3: control (artificial saliva at pH 7). Each subgroup comprises 14 teeth from each group tested at different time points. Subgroup 1: (immediately after bonding), Subgroup 2: (after 6 days of bonding), Subgroup 3: (after 12 days of bonding)

Time points	ARI Scores	Group 1(*n* = 42)	Group 2 (*n* = 42)	Group 3(*n* = 42)
		**n (%)**		
Subgroup 1	Score 0	2 (14.3%)	1 (7.1%)	1 (7.1%)
	Score 1	7 (50%)	6 (42.9%)	6 (42.9%)
	Score 2	4 (28.6%)	6 (42.9%)	6 (42.9%)
	Score 3	1 (7.1%)	1 (7.1%)	1 (7.1%)
Subgroup 2	Score 0	1 (7.1%)	1 (7.1%)	1 (7.1%)
	Score 1	7 (50%)	6 (42.9%)	7 (50%)
	Score 2	6 (42.9%)	5 (35.7%)	6 (42.9%)
	Score 3	0 (0%)	2 (14.3%)	0 (0%)
Subgroup 3	Score 0	0 (0%)	1 (7.1%)	2 (14.3%)
	Score 1	8 (57.1%)	3 (21.4%)	5 (35.7%)
	Score 2	4 (28.6%)	6 (42.9%)	6 (42.9%)
	Score 3	2 (14.3%)	4 (28.6%)	1 (7.1%)

## Discussion

Swimming is a widely practiced sport around the globe. Several studies [2–4] have explored the effects of swimming pool water on human dental health; however, there remains a significant gap in research regarding its impact on the bond between orthodontic brackets and human teeth. The present study aimed to investigate this critical issue.

The Findings indicated that the bond strength of teeth soaked in chlorinated water with a pH of 3 was significantly lower than that of teeth soaked in chlorinated water with a pH of 7.4 or those in the control group. This suggested that the increased acidity might have led to greater dental erosion, thereby compromising the bond strength between the teeth and the orthodontic brackets. The differences observed across the three groups were statistically significant, leading to a rejection of the null hypothesis.

In the present study, the teeth were randomly allocated into three experimental groups, each representing a distinct exposure condition. The first group was exposed to chlorinated water with a pH of 7.4 (representative of well-maintained swimming pool water) according to the Centers for Disease Control and Prevention (CDC) guidelines for swimming pool water regulation [26]. The second group was exposed to chlorinated water with a pH of 3, reflecting the conditions found in poorly monitored swimming pools. This particular pH was chosen on the basis of the findings of Abdelrahman et al., who suggested that insufficient buffering in pools can lead to a rapid decrease in pH [2].The control group comprised teeth soaked in artificial saliva, which has been established in previous studies not to alter the enamel structure [27–29].

The pH of swimming pools can fluctuate due to several factors, including weather, water temperature, swimmer activities, and chemical evaporation. These variables necessitate daily testing of the pool pH. In the present study, a fresh solution was prepared every day to maintain the pH and ensure the stability of the experimental conditions [30].

The thermocycling procedure employed in this study effectively simulates the aging process of dental samples, mimicking the temperature changes in the oral environment that exert stress on orthodontic brackets. Various studies [21, 23, 31, 32] have utilized different methodologies for thermocycling; however, Gale et al. recommended using 10,000 cycles in water ranging from 5 °C and 55 °C, with a dwell time of 30 s and a transfer time of 5 s, as the most suitable approach for representing one year of aging [23].

The results indicated that the SBS was significantly lower in the experimental groups than in the control group. This reduction may be attributed to pH-induced dental erosion [2, 4], leading to enamel weakness and, subsequently, a decrease in bond strength. Research by Engineer et al. [33] supports these findings, indicating that swimmers frequently exhibit symptoms of dental erosion, such as chalky enamel, increased surface roughness, and heightened sensitivity. Similar results were reported by Wang et al. [11], who reported that erosion from acidic beverages decreased the SBS. Additional studies by Pasha et al. [34], Casas-Apaycoet al. [13], and Hosni et al. [14] highlighted the detrimental effects of carbonated drinks on enamel and bond strength. Enamel erosion results in the degradation of the bonding substrate between teeth and brackets, adversely affecting the adhesive material. In Group 1, despite the neutral pH, a decrease in SBS was noted, possibly due to prolonged exposure of the composite adhesive to chlorinated water, which might affect its properties and contribute to the wear of the composite, thus influencing the bond strength of the brackets [35]. Further investigation in this respect is needed.

The results demonstrated a decrease in the SBS across the test groups from the initial bonding assessment through days 6 and 12. Prolonged exposure to chlorinated water might have intensified the severity of dental erosion, resulting in a progressive decline in SBS over time. These findings were consistent with those of Abdelrahman et al. and Favero et al. [2, 36], who reported that an increase in immersion duration correlated with a greater incidence of eroded samples. Abdelrahman et al. [2] reported that dental erosion rates were 26% for competitive and 10% for noncompetitive swimmers. The extended training time for competitive swimmers leads to greater exposure to chlorinated water, exacerbating its detrimental effect on dental enamel. The authors concluded that factors such as swimming duration, the frequency of training, and the water pH significantly influence dental erosion. This conclusion contrasts with the findings of Santos et al. [16], who suggested that immersion in Coca-Cola resulted in greater bond strength than lime and saliva did and indicated that immersion duration did not affect SBS. On the other hand, Lenzi et al. [12] reported that enamel erosion increased bond strength, suggesting that porosities from erosion enhance mechanical interlocking between the enamel and adhesive.

The adhesive remnant index is commonly employed to assess the amount of adhesive residue remaining on the enamel surface postdebonding [37]. In the present study, the ARI scores did not significantly differ across the three groups, with the majority of the scores being 1 or 2, indicating adhesive and cohesive failure. Adhesive failure might be attributed to enamel weakness due to dental erosion induced by the acidic nature of chlorinated water, whereas cohesive failure might have resulted from the degradation of the composite adhesive in an acidic environment. Soleman et al. and Tussi et al. [38, 39] reported that acidic water could increase water sorption in composite resins, leading to hydrolysis and dissolution of their components. However, the effectiveness of the ARI in accurately reflecting bond strength is debatable, as it is determined subjectively by operator evaluation [40–47].

### Limitations of the study

1. Despite daily preparation and continuous monitoring of the solutions, their pH cannot be fully controlled because of various factors such as temperature and humidity.
2. The study utilized only one type of tooth (premolars) for ease of collection.
3. The in vitro nature of the study does not fully simulate the oral environment, which may differ because of the presence of saliva and the composition of ingested food and beverages.

## Conclusion

Within the limitations of this study:

1. The pH of chlorinated swimming pool water affects the bond strength of orthodontic brackets. Accordingly, continuous monitoring of the pH of swimming pool water is essential.
2. The more acidic pH of the chlorinated water results in a lower bond strength between the orthodontic brackets and the teeth.
3. The type of bond failure is not affected by the pH of chlorinated water, with failures predominantly presenting as adhesive and cohesive failures.

### Recommendations

1. the pH of the swimming pool was continuously monitored.

2. a scanning electron microscope was used to investigate the effects of exposure to chlorinated water on the enamel surface.

3. Investigate the effect of chlorinated water on the physical and mechanical properties of clear aligners.

## Data Availability

The datasets used during the current study are available from the corresponding author upon reasonable request. All the data analyzed during this study are included in this published article in the form of tables and figures.

## Abbreviations

SBS: Shear bond strength
ARI: Adhesive remnant index
